# Community health workers at the dawn of a new era: 7. Recent advances in supervision

**DOI:** 10.1186/s12961-021-00754-6

**Published:** 2021-10-12

**Authors:** Carey Westgate, David Musoke, Lauren Crigler, Henry B. Perry

**Affiliations:** 1 Community Health Impact Coalition, New York, NY USA; 2 Department of Disease Control and Environmental Health, School of Public Health, College of Health Sciences, Makerere University, Kampala, Uganda; 3 Crigler Consulting, LLC, Hillsborough, NC USA; 4 Department of International Health, Health Systems Program, Bloomberg School of Public Health, Johns Hopkins University, Baltimore, MD USA

**Keywords:** Community health, Community health workers, Supervision, Supportive supervision, Supervision approaches, Performance management

## Abstract

**Background:**

Supervision is essential for optimizing performance and motivation of community health workers (CHWs). This paper, the seventh in our series, “Community health workers at the dawn of a new era”, supplements the existing evidence on CHW supervision in low- and middle-income countries by reviewing what supervision approaches are employed in specific contexts, identifying potential facilitators of CHW supervision including mobile health (mHealth) interventions, and noting challenges of supervision including the relationship between supervision and other CHW programme elements.

**Methods:**

For this exploratory research study on CHW supervision, we reviewed the supervisory interventions described in a compendium of 29 case studies of large-scale CHW programmes, performed an electronic search of multiple databases to identify articles related to CHW supervision published between 15 June 2017 and 1 December 2020, and from those articles followed additional references that appeared to be relevant for our results.

**Results:**

We reviewed 55 case studies, academic articles, and grey literature resources as part of this exploratory research. A variety of supervision approaches have been adapted over time, which we grouped into five categories: external supervision, community supervision, group supervision, peer supervision, and dedicated supervision. These approaches are frequently used in combination. Digital (mHealth) technologies are being explored as potential facilitators of CHW supervision in both small- and large-scale programmes; however, evidence of their effectiveness remains limited to date. Inadequate support for supervisors is a major challenge, particularly given the numerous and varied roles they are expected to fulfil, spanning administrative, clinical, and supportive activities. Supervisors can help CHWs acquire other critical elements needed from the health system for them to perform more effectively: incentives to foster motivation, clarity of roles and tasks, adequate tools and supplies, appropriate knowledge and skills, and a safe work environment.

**Conclusion:**

In the absence of a universal “best approach” for CHW supervision, our recommendation is that countries and programmes prioritize homegrown evolution over time to suit the local context. In some cases, this may involve scaling up novel approaches that have proven effective at small scale or testing approaches that have worked in other countries.

**Table:** 

Key messages box 1: Summary	
**Key findings**	
We conducted exploratory research of the academic literature and grey literature on supervision of community health workers (CHWs) in low- and middle-income countries, published after 15 June 2017. We concluded that: • Supervisors in CHW programmes are expected to perform a variety of roles covering administrative, clinical, and supportive activities • Multiple supervision approaches are in use today: external supervision, community supervision, group supervision, peer supervision, and dedicated supervision • Inadequate support for supervisors is a major challenge. With the right support, supervisors can help CHWs acquire and obtain other elements needed for strong performance, such as pay/incentives, role clarity, tools/supplies, and knowledge	
**Key implications**	
• CHW programmes now have the opportunity and the necessity for instituting stronger supervision as one of the critical steps in moving to a higher level of performance • Increased, dedicated funding from national governments with support from the international community will be required to try new approaches, scale up existing approaches deemed promising, and evaluate the quality and effectiveness of such approaches alongside other health systems improvement initiatives	

## Background

Harnessing the potential of community health worker (CHW) programmes has been identified as a pathway to strengthen primary healthcare (PHC) and the health workforce [1], thereby advancing the goals of achieving universal health coverage (UHC) and ending preventable maternal and child deaths by 2030 [2, 3]. CHWs can improve population health and contribute to global health goals [4–7], but require conducive health policies and system support, including supervision, in order to achieve their full potential [1].

Supervision (also referred to as “supportive supervision”) is considered an essential element of effective CHW programmes and is defined as a “process of guiding, monitoring, and coaching workers to promote compliance with standards of practice and assure the delivery of quality care service” [8]. Supervision promotes quality at all levels of the health system by strengthening relationships among different actors, focusing on identification and resolution of problems, helping to optimize allocation of resources, and promoting standards, teamwork, and better two-way communication [9].

Traditional supervisory roles include reviewing data collected by CHWs and submitting it to higher levels of the health system; conducting quality assurance activities such as clarifying the role and tasks of the CHW and performing service delivery observations; reviewing CHW record-keeping; and offering coaching and problem-solving if needed. Problem-solving can include helping the CHW restock supplies, ensuring incentives are paid in full and on time, asking questions, troubleshooting challenges encountered in the field, and conveying challenges to higher-level decision-makers [8]. CHW supervisors are also expected to play a supportive role. This includes providing counselling and support to CHWs; helping CHWs build trusted relationships with community members; connecting the CHW with continued professional development opportunities; facilitating career advancement pathways; and reaffirming the importance and details of the CHWs’ role in the community [10]. All of these activities are intended to improve the motivation and performance of CHWs.

Despite widespread recognition of the importance of adequate supervision of CHWs, the evidence related to what supervision approaches are most effective and in what context is less clear [1]. The multitude of approaches to CHW supervision, and the fact that the supervisory role is carried out by a variety of actors in different programmes and contexts [8], makes it challenging to compare supervisory effectiveness, costs, acceptability, feasibility, and other outcomes across programmes [1]. Consequently, the 2018 WHO Guideline recommendations on CHW supervision strategies, supervision strategies, based on a systematic review of published reviews [11] published between 1 January 2005 and 15 June 2017, were made on a *conditional* basis, noting that “very limited information was available to compare specific supervision strategies” [1].

**Table:** 

Key message box 2	
Despite widespread recognition that adequate supervision of CHWs is important, the 2018 WHO Guideline for optimizing CHW programmes found insufficient evidence on which supervision approaches are most effective and in what context	

However, new evidence related to supervision of CHWs, including a compendium of case studies of large-scale CHW programmes and the details of how supervision is implemented in each [12], has been published since the release of the WHO Guideline. On the basis of this new evidence, we undertook exploratory research using academic and grey literature published after 15 June 2017 (cut-off date for the WHO review) in order to answer the question of what supervision approaches are most effective and in what context. Based on the findings of our exploratory research, we discuss implications for policy-makers, programme managers, researchers, and other stakeholders seeking to optimize CHW supervision and propel CHW programmes across the threshold into a new era in which their contributions to health systems are greatly enhanced.

## Methods

### Study design

We conducted exploratory research and mapped available literature on specific topics related to supervision of CHWs. In addition to reviewing the supervisory model described in each of 29 large-scale CHW programme case studies published in 2020, we searched multiple databases and then applied a snowball sampling technique to identify relevant articles on CHW supervision.

### Inclusion criteria

Our exploratory research included a review of programme case studies, journal articles, and grey literature on CHW supervision in low- and middle-income countries (LMICs), published after 15 June 2017. This cut-off date was selected because it was the end date for the search utilized in the systematic review of reviews for the WHO Guideline [1]. Discussion papers, protocols, and conference abstracts were excluded.

### Search strategy and data sources

For the database search, we utilized keyword search terms summarized in Table 1. This search strategy or a modification thereof was carried out in PubMed, EMBASE, CINAHL Plus, Web of Science, and CHW Central.

**Table 1:** Search terms and strategy

Database	Search terms
PubMed	Query
Search #3	Search (#1 AND #2)
Search #2	Search (("supervision"[ti] OR "supervisor")[ti] OR ("performance management"[ti] OR "human resource management")[ti])
Search #1	Search(“community health workers”[tiab] OR “lay health workers”[tiab] OR “volunteer health workers”[tiab] OR “community health promoters”[tiab] OR “village health workers”[tiab] OR “village health volunteers”[tiab] OR “lady health workers”[tiab] OR “community health aides”[tiab] OR “home based carers”[tiab] OR “community health agents”[tiab] OR “health surveillance assistants”[tiab] OR “traditional birth attendants”[tiab])

### Study selection

Carey Westgate (CW) reviewed the supervision details of 29 programme case studies contained in the 2020 compendium [12] and extracted relevant details for analysis. CW designed and executed the database search strategy on CHW supervision, removed duplicates, reviewed titles and abstracts of articles to determine which articles met the inclusion criteria, and extracted relevant details in an Excel workbook. To supplement the database search, Henry Perry (HP), Lauren Crigler (LC), David Musoke (DM), and CW reviewed the reference lists of included articles to purposively identify additional resources on CHW supervision that met the inclusion criteria. For these articles, CW extracted relevant details in an Excel workbook.

### Data extraction

The following data from the included case studies, articles, and grey literature were extracted: (1) general characteristics of the study, (2) study summary, (3) facilitators of CHW supervision, and (4) challenges related to supervision of CHWs. General characteristics included a regional focus, study type, and CHW programme scale (national versus subnational). The study summary consisted of the specific research question or objective, outcomes evaluated, the definition of supervision (if provided), a description of the supervision intervention, and the main findings. Facilitators of CHW supervision included tools or recommended practices including mobile health (mHealth) interventions. Challenges were both specific to supervision itself and contextual health system factors potentially impacting supervision.

### Data synthesis

We used the extracted data from the 29 case studies and included articles to generate results around what approaches to supervision are commonly practiced and in what contexts. For the 29 case studies of large-scale programmes, we categorized the dominant supervision approach(es) according to one of five (not mutually exclusive) approaches, described below. While we did not systematically review the other included articles according to the five supervision approaches, we did extract relevant findings from these articles to inform our description of each approach and provide more detail about the approach “in action”. These articles also informed our discussion of the effectiveness and challenges associated with each approach, as well as cross-cutting facilitators and challenges relevant across all approaches.

## Results

### Characteristics of included studies

A total of 55 studies were reviewed as part of this exploratory research. Tables 2 and 3 contain characteristics of the included studies. Twenty-nine of the 55 studies were individual case studies derived from a single compendium that describes national CHW programmes from multiple perspectives: historical context, health needs, health system structure, CHW programme features, scope of work, selection and training, support and supervision, incentives and remuneration, community role, linkages with the formal health system, programme scale-up, monitoring and data use, financing, impact, and challenges [12]. These individual case studies are cited in the References [13–41]. An additional 26 studies were specifically about supervision in CHW programmes: 16 were identified through the database search and an additional 10 were identified by consulting the reference lists of articles in our database search results.

**Table 2:** Characteristics of included studies

Descriptor	Count of included studies		
	Case studies from compendium	Academic articles identified from literature search	Total
Study type			
Case study	29	0	29
Descriptive/qualitative	0	15	15
Experimental	0	7	7
Expert opinion/practitioner resource	0	2	2
Review	0	2	2
Publication date			
2017 (after 15 June 2017)	0	4	4
2018	0	7	7
2019	0	11	11
2020	29	3	32
2021	0	1	1
Regional focus			
Africa	16	19	35
South-East Asia	8	0	8
Eastern Mediterranean	3	2	5
Americas	2	0	2
Multiple	0	5	5
Total	29	26	55

**Table 3:** List of included studies

First Author	Date	Study type	WHO region	Country	Reference number in bibliography
Aftab	2018	Experimental	Eastern Mediterranean	Pakistan	[66]
Agarwal	2019	Review	Multiple	Various LMICs	[68]
Aitken	2020	Case study	Eastern Mediterranean	Afghanistan	[13]
Assegaai	2021	Descriptive/qualitative	African	South Africa	[49]
Assegaai (A)	2019	Descriptive/qualitative	African	South Africa	[44]
Assegaai (B)	2019	Descriptive/qualitative	African	South Africa	[67]
Ban	2020	Case study	South-East Asia	Nepal	[30]
Biemba (A)	2020	Descriptive/qualitative	African	Zambia	[62]
Biemba (B)	2020	Experimental	African	Zambia	[61]
Biemba	2017	Descriptive/qualitative	African	Zambia	[60]
Brieger	2020	Case study	African	Nigeria	[32]
Damtew	2020	Case study	African	Ethiopia	[17]
Davis	2020	Case study	South-East Asia	Myanmar	[29]
Davlantes	2020	Case study	African	Mozambique	[28]
Dyson	2020	Case study	African	Sierra Leone	[35]
Fundira	2020	Case study	African	Zimbabwe	[41]
Gaudrault	2017	Expert opinion	Multiple	Various LMICs	[45]
George	2020	Experimental	African	South Africa	[69]
Giugliani	2020	Case study	Americas	Brazil	[16]
Joardar (A)	2020	Case study	South-East Asia	Bangladesh (government programme)	[14]
Joardar (B)	2020	Case study	South-East Asia	Bangladesh (BRAC programme)	[15]
Kim	2019	Descriptive/qualitative	African	Uganda	[65]
Koffi	2020	Case study	African	Madagascar	[26]
Kok	2018	Descriptive/qualitative	African	Ethiopia, Kenya, Malawi, Mozambique	[46]
Lassi	2020	Case study	Eastern Mediterranean	Pakistan	[33]
Lazzerini	2019	Experimental	African	Uganda	[59]
Ludwick	2018	Descriptive/qualitative	African	Uganda	[55]
Millogo	2019	Descriptive/qualitative	African	Burkina Faso	[57]
Musoke	2019	Descriptive/qualitative	African	Uganda	[54]
Musoke	2020	Case study	African	Uganda	[39]
Mwinnyaa	2020	Case study	African	Ghana	[18]
Napier	2020	Case study	African	Rwanda	[34]
Njiraini	2020	Case study	African	Kenya	[24]
Nsibande	2018	Descriptive/qualitative	African	Ethiopia	[56]
O'Connor	2019	Experimental	African	Sierra Leone	[47]
Oendari	2020	Case study	South-East Asia	Indonesia	[22]
Phiri	2017	Descriptive/qualitative	African	Zambia	[64]
Pongpirul	2020	Case study	South-East Asia	Thailand	[38]
Rahbar	2020	Case study	Eastern Mediterranean	Iran	[23]
Rahman	2019	Experimental	Eastern Mediterranean	Pakistan	[63]
Rogers	2020	Case study	African	Liberia	[25]
Rosales	2020	Case study	Americas	Guatemala	[19]
Rowe	2018	Review	Multiple	Various LMICs	[58]
Schneider	2020	Case study	African	South Africa	[36]
Schwarz	2019	Descriptive/qualitative	African	Ghana	[42]
Scott	2020	Case study	South-East Asia	India	[20]
Shelley	2020	Case study	African	Tanzania	[37]
Simkoko	2020	Case study	African	Malawi	[27]
SPRING	2017	Expert opinion	Multiple	Various LMICs	[70]
Strodel	2020	Case study	African	Niger	[31]
Strodel	2020	Case study	South-East Asia	India	[21]
Tseng	2019	Descriptive/qualitative	African	South Africa	[43]
Vallières	2018	Descriptive/qualitative	Multiple	Sierra Leone, Bangladesh, Ethiopia, Indonesia, Kenya, Malawi, Mozambique	[71]
Whidden	2018	Experimental	African	Mali	[50]
Wilmink	2020	Case study	African	Zambia	[40]

The full reference can be found in the bibliography

*BRAC* Building Resources Across Communities

### Approaches for CHW supervision

The CHW Reference Guide, published in 2014, described four distinct approaches to CHW supervision—supervision by facility-based workers, community supervision, group supervision, and peer supervision—that may be appropriate in different contexts [8]. Based on our review of CHW supervision described in case studies and academic literature published since 15 June 2017, we added a fifth approach to this list: dedicated supervision. In Table 4 below, we summarize the dominant supervision approach(es) employed by 29 large-scale CHW programmes, across 27 countries, according to these five approaches. Some programmes employ these approaches in combination. In the subsections that follow, we define each approach, provide examples of large-scale programmes adopting this approach, and discuss evidence for the effectiveness of each approach as well as any challenges based on our review of the literature published since 15 June 2017.

**Table 4:** Supervision approach(es) employed in large-scale CHW programmes

Country	Facility/district	Group	Community	Peer	Dedicated	Dominant supervision approach
Afghanistan	X	X				Each health facility supporting health posts has a community health supervisor (CHS) that visits health posts to support and supervise the CHWs. In addition, the CHWs come monthly to the supporting health facility for a joint meeting with the other CHWs. During these encounters, the CHWs receive continuing education and have an opportunity to discuss problems encountered in day-to-day work [13]
Bangladesh (Government CHW programme)					X	Family welfare assistants (FWAs) are supervised by male supervisors, called family planning inspectors, with whom they meet at least twice per month during field visits. Health assistants (HAs) are supervised by assistant health inspectors, each of whom is responsible for three HAs (one in each ward) [14]
Bangladesh (BRAC NGO CHW programme)	X			X (dual-tier)		Direct supervision of *shasthya shebikas* (SSs) is conducted by *shasthya kormis* (SKs). SKs are supervised by BRAC area managers [15]
Brazil	X					Community health assistants (CHAs) are supervised by nurses and physicians from the family health teams based at the local clinics with which they are attached. The supervision process varies among family health teams depending on capabilities and needs [16]
Ethiopia	X		X	X (dual-tier)		Health extension workers (HEWs) are primarily supervised by the health centre staff, who conduct regular supportive supervision visits to improve the capacity of HEWs to provide health services to the community. The village health committee and community members are also very involved in supporting the HEWs and evaluating their performance. HEWs provide supervision and support to Women’s Development Army volunteers [17]
Ghana	X		X	X (dual-tier)		Community health officers (CHOs) are supervised monthly by public health nurses, physician assistants, and subdistrict community health planning and services coordinators. CHOs supervise community health volunteers (CHVs) monthly. Community health management committees also supervise the work of the CHVs [18]
Guatemala (Government CHW Program, closed in 2013)	X					CHWs were supervised by the mobile health team. CHWs also reported directly to a *técnico en salud rural* (rural health technician) who was under the supervision of a physician or professional nurse [19]
India (current CHW programme)	X					Accredited social health activists (ASHAs) report to ASHA facilitators, and anganwadi workers (AWWs) report to anganwadi supervisors (*mukhya sevikas*) [20]
India (defunct Village Health Guides programme)	X		X			There was no formal supervision. Village health guides went to the PHC centre to collect their salaries and connect with the staff there [21]
Indonesia	X		X			While the nearest community health centre (*Puskesma*) provides technical guidance and support, the real accountability of the CHWs (*kaders)* is to the village committee that appointed and supports them in their work [22]
Iran	X					Higher-level staff, including those from rural and urban centres of comprehensive health services and district health centres, as well as the deputy for health at universities of medical sciences, make regular supervisory visits to health houses and health hosts. University professors and faculty from the University of Medical Sciences evaluate programme effectiveness and quality, and then they make decisions about needed programme revisions [23]
Kenya	X					Each CHV should receive supportive supervision monthly from a community health extension worker (CHEW), either at the health facility or in the community. The supervision consists of training, review of reports, and household visits with a CHEW. CHEWs follow a checklist to ensure quality supervision [24]
Liberia					X	Each community health service supervisor (CHSS) supervises approximately 10 CHAs. The CHSS is a new cadre. Each CHSS has already been trained as a health worker (nurse, midwife, or physician’s assistant) and receives an additional four weeks of training. Supervision occurs both in the field and during monthly meetings at the nearest health facility [25]
Madagascar	X					*Agents communautaires* (ACs) are supervised by the head of the peripheral basic health centres (*chef centre de santé de base*), while *agents communautaires nutritional* (ACNs) report to an animator, who is a supervisor employed by a local NGO that has been contracted by the National Nutrition Office to provide nutrition services. There was no formal linkage between ACNs and Ministry of Health (MOH)-operated basic health centres until 2019 [26]
Malawi	X			X (dual-tier)		Health surveillance assistants (HSAs) are supervised on a monthly basis by a senior HSA and then on a quarterly basis by an assistant environmental health officer, an environmental health officer, or a community health nurse. HSAs themselves supervise other community-level cadres such as traditional birth attendants and village health and water committees [27]
Mozambique	X					A staff member at the nearest health facility supervises all *agentes polivalentes elementares* (APEs) in the facility’s catchment area. Supervisors meet with APEs on a monthly basis to review register books and distribute new commodity kits. They also make periodic supervisory visits to APEs in their communities [28]
Myanmar	X					Community-based health worker (CBHW) supervision varies by programme for: HIV, malaria, tuberculosis, newborn/child health, and reproductive health, but generally relies on facility-based staff [29]
Nepal	X	X		X (dual-tier)		Most FCHVs visit their respective health facility every month; there, they receive supplies, materials, commodities, and programmatic advice and feedback. Supervision is done by auxiliary health workers [30]
Niger	X			X (dual-tier)		Each health post is linked to a health centre. A staff member of the health centre is supposed to supervise *agents de santé communautaire* (ASCs), but this is infrequent and irregular. ASCs supervise *relais* volunteers [31]
Nigeria	X	X				Supervision of volunteer village health workers (VHWs) is variable and often involves NGO staff. CHEWs are supervised by the person in charge of the closest health facility. VHWs are supervised by CHEWs in group monthly meetings [32]
Pakistan					X	Supervision is highly organized and multitiered. Lady health workers (LHWs) are each attached to a public health clinic and are supervised by an LHW supervisor. One LHW supervisor is responsible for supervising 25 LHWs. LHWs should have community-based supervision at least once a month, at which time the LHW supervisor meets with clients and with the LHW, reviews the LHW’s work using a structured monitoring checklist, and makes a work plan for the next month [33]
Rwanda	X			X		CHWs are supervised directly by the health centre with the support of volunteer cell coordinators [34]
Sierra Leone	X			X	X	Peer supervisors were formally introduced in 2016. Peer supervisors are full-time supervisors. They do not provide CHW services in addition to their supervisory roles. Peer supervisor selection prioritizes those who have previously served as CHWs and have been identified as high-performing leaders among the group of CHWs linked to a peripheral health unit. In addition to data collection and reporting, their role focuses on coaching, mentorship, and on-the-job training of the CHWs within their catchment area [35]
South Africa	X				X	Ward-based primary healthcare community outreach teams (WBPHCOTs) are supervised by outreach team leaders, who are higher-level (professional) or mid-level nurses specifically appointed or seconded from local PHC facilities. Teams refer clients and report to designated PHC facilities, which are also supposed to provide supplies and space for the outreach teams [36]
Tanzania	X		X			The village health committee is responsible for overall supervision of the community-based health programme within the village catchment, including implementation of village health plans, mobilizing resources, compiling reports, and supervising the nominee and application process. CHWs receive administrative supervision from village executive officers, to whom they report daily. CHWs receive periodic on-the-job supportive supervision and training on clinical tasks from the in-charge/focal persons at the health dispensary or health centre to which the CHW is attached [37]
Thailand	X					The village health volunteer (VHV) programme is under the primary healthcare division of the Department of Health Service Support of the MOH. VHVs are supervised by on-site local health workers. A web-based VHV information platform has been developed and is widely used, and a cell phone application has been introduced [38]
Uganda	X					Village health teams (VHTs) are supposed to report to a health facility within their community where a health worker supervises them. A parish coordinator often offers support to all VHTs within a parish, and the district health educator is mandated to oversee the work of all VHTs in the district. Due to limited funding and human resource capacity, supervision of VHTs is often irregular and inconsistent [39]
Zambia	X					CHAs are supervised by a skilled health worker who is the officer-in-charge at the health facility to which they are linked. Supervision is supposed to be conducted at the health facility and the community level on a monthly basis using standardized supervisory checklists, but is often not carried out because of the pressing clinical demands of the supervisors [40]
Zimbabwe	X		X			VHWs are directly supervised by the nurse-in-charge at the nearest health centre within their ward. They report monthly and quarterly to their local health centre. They are also broadly supervised and supported by community leaders and the ward health team [41]
Number of case studies employing this approach	26 out of 29	3/29	6/29	8/29	5/29	

“Dual-tier” supervision refers to one cadre of CHWs supervising a lower-level CHW cadre

*BRAC* Building Resources Across Communities, *NGO* nongovernmental organization

### Supervision by district or health facility staff

The most common approach to CHW supervision in large-scale programmes is to employ district or facility-based staff as supervisors; this approach was used in 26 out of the 29 case studies examined [12]. Facility-based supervisors cover a broad swath of health worker cadres based in facilities, administrative staff from other levels of the health system, and occasionally external (i.e., NGO) staff. It is often combined with other approaches, most commonly community-based supervision (Table 4).

This approach promotes strong clinical oversight, coaching, and mentoring of CHWs; incorporation of new protocols and procedures into CHW workflows; and better overall integration with the health system, including supply chain functions [10]. There is some debate over what type of facility staff member from what type of facility is best suited to play the supervisory role, with implications for costing of supervision. On this topic, the evidence is mixed. In a study from Ghana, the type of supervisor (i.e., community health officers, public health nurses, midwives, health assistants, and physician assistants) and type of facility (i.e., community health post, health centre, or hospital) to which the CHW was linked had no impact on the frequency of supervision, though the quality of supervision was not assessed [42]. Meanwhile, a qualitative study in South Africa concluded that supervision by senior nurses in facilities promoted better integration with the facility teams compared to supervision by a junior nurse [43].

A concern with CHW supervision by facility-based staff is that facility-based supervisors often already shoulder a heavy workload at the facility and may lack the time to provide supportive supervision to CHWs. This may result not only in reduced frequency and lower quality of supervisory visits [12], but also in strained relationships between supervisor and supervisee [44].

### Community supervision

Community supervision involves community groups, health committees, or community associations monitoring CHW performance and providing feedback [10]. This approach was reported in six out of the 29 case studies of large-scale programmes (Ethiopia, Ghana, India, Indonesia, Tanzania, Zimbabwe) and in all cases was used in combination with the facility-based approach (Table 4). There is a long legacy of community-based supervision. In 1989, WHO recommended that CHW programmes receive support of a group of community members with active links to the health sector [45]. These groups—which go by many names such as village health committees or community health committees—not only provide support to CHWs but also assess and track local health issues, mobilize communities to address issues, and advocate for improved health services [45].

Community-based supervisory groups can step in where facility-based supervisors are unable due to competing priorities at the health facility or sociocultural barriers. While promising, community supervision is not applicable to all programmes, as it works best where strong community structures are already active in community management [10]. Additionally, community-based structures require their own supervision in order to be successful; these are outlined in the 2017 programme functionality assessment for community and health facility management committees [45].

### Group supervision

Group supervision involves a group of CHWs meeting together with a supervisor at health centres or in villages, where they collectively perform regular supervisory activities such as collecting data, discussing problems, and continuing education [10]. This approach was documented in three of the 29 large-scale programmes considered in our exploratory research (Afghanistan [13], Nigeria [32], and Nepal [30]), although it is likely to be much more commonly utilized. In all three cases, group supervision was used in combination with the facility/district supervisor approach (Table 4).

A mixed-methods study of a group supervision intervention in four countries found that group supervision improved CHW motivation by providing a forum for CHWs to be recognized, feel supported, gain knowledge, and work as a team [46]. A potential drawback of this approach is the lack of individualized, personalized feedback in a group setting, although this can be mitigated by using group supervision in combination with other approaches, as is the case for volunteer village health workers (VVHWs) in Nigeria [32].

### Peer supervision

Peer supervision is defined as “CHWs helping other CHWs learn new skills and assessing the quality of work performed by fellow CHWs” [8]. This approach was practiced in eight out of the 29 case studies (Bangladesh—BRAC, Ethiopia, Ghana, Malawi, Nepal, Niger, Rwanda, Sierra Leone) included in our exploratory research (Table 4). In all eight case studies, peer supervision was employed alongside supervision by facility/district-based staff. Peer supervision has also been analysed in combination with other approaches in formal studies of supervision. For example, a mixed-methods study in Ethiopia, Kenya, Malawi, and Mozambique found that peer supervision in combination with group supervision encouraged teamwork, coordination of tasks between CHWs and other health professionals, and increased accountability to each other and to the organization [46]. Particularly when implemented alongside community engagement strategies, the use of CHW peer supervisors shows potential [47].

Other advantages of peer-based supervision include an enhanced mutual understanding between supervisors and CHWs of the challenges that CHWs face in the field, as well as career advancement opportunities for high-performing CHWs who can take on new supervisory responsibilities [12].

Peer supervision is often embedded in dual-tier systems, in which a paid, higher-level CHW supervises one or more lower-level volunteer CHWs, and volunteers work together [48]. In some of these systems (such as in Sierra Leone), high-performing peers are often promoted to a more formal supervisory role. Examples include Ethiopia’s health extension workers and Health Development Army volunteers, Ghana’s community health officers and community health volunteers, Niger’s *agents communautaires* and *relais*, and Nepal’s auxiliary health workers and female community health volunteers [12] (Table 4).

### Dedicated supervision

The idea that supervisors should exclusively focus full-time on managing CHWs—rather than assuming this task in addition to existing clinical and nonclinical duties—is important for “dedicated” supervision, a trend that has emerged in recent years [12]. Dedicated supervision may improve integration between CHWs and the health system, especially at the PHC facility level. For example, in South Africa, dedicated “outreach team leaders” (OTLs) were seen as providing a necessary layer of supervisory support between CHWs and the PHC facilities to which they are linked; in the absence of dedicated team leaders, facility staff were less clear about CHWs’ roles and their own role in providing support to CHWs [49]. Dedicated supervision has also been linked to improved CHW performance and health outcomes. In Mali, a “360 Supervision Approach” in which dedicated CHW supervisors provide monthly individual supervisory sessions (lasting approximately three hours) along with weekly group supervisory sessions (lasting approximately two hours) has been shown to improve CHW performance over time on key integrated community case management (iCCM) indicators related to quantity, quality, and timeliness of care [50].

Although most examples of dedicated CHW supervision are in small-scale programmes, there are exceptions. We identified the dedicated supervision approach in five out of the 29 case studies (Bangladesh, Liberia, Pakistan, Sierra Leone, South Africa) included in our exploratory research (Table 4). As documented in the Liberia CHW programme case study [25], following the 2013–2016 Ebola epidemic, Liberia established a cadre of dedicated, full-time supervisors known as community health service supervisors (CHSSs). CHSSs are recruited as professionally trained health workers, and each is responsible for supervising 10 CHWs. CHSSs are expected to spend 80% of their time in the field performing supportive supervision visits to each of their assigned CHWs [25]. We were unable to find any studies on the effectiveness of this approach in Liberia.

As noted above, South Africa’s ward-based primary healthcare outreach team (WBPHCOT) model also incorporates elements of the dedicated supervision approach. WBPHCOTs are supervised by Outreach Team Leaders (OTLs), typically a nurse, who is expected to devote 70% of his or her time outside the facility to providing supervision support and evaluation for CHWs in the field. Unfortunately, this example of dedicated supervision—which contributed to the programme being deemed effective in a previous evaluation [51]—has struggled to sustain initial successes due to fiscal and political challenges [49]. A shortage of nurses and the generally high workload faced by OTLs mean that time which OTLs should be dedicating to CHW supervision is instead spent on other tasks [36]. In at least one province, failure to renew the contracts of OTLs contributed to major shortages in supervisory personnel and undermined early successes achieved in the WBPHCOT programme [49].

**Table:** 

Key message box 3	
A relatively new and promising approach to CHW supervision involves the use of “dedicated” supervisors who are exclusively focused on oversight of CHWs without the additional burden of facility-based, clinical duties. Formal evaluations of this approach are needed	

### Facilitators of CHW supervision

Supervision plans and guidelines, supervisor job descriptions and qualifications, supervision checklists and job aids, supervision reports, supervision of supervisors, and supervision training documents can facilitate CHW supervision [52, 53]. Additionally, ensuring that supervisors have access to transportation is essential for monthly field visits to collect reports and distribute medicines [54]. Several studies have shown that close relationships with supervisors can motivate CHWs and improve performance, suggesting that relationship-building between supervisor and supervisee can be another facilitator of supervision [46, 55, 56]. Cascade approaches that assign supervisors of supervisors, promote integration with the health system, and delineate supervisory roles at various tiers of the health system also show promise [57].

"Multifaceted strategies" which combine strengthened infrastructure, financing, supervision, management techniques, and training have been associated with greater improvements in health worker performance compared to interventions implemented in isolation [58]. For example, an intervention combining small financial incentives for CHWs with supportive supervision proved to be effective in encouraging care-seeking behaviour by caregivers of malnourished children [59].

Several studies in smaller-scale programmes have explored the potential of mHealth interventions to facilitate the CHW supervision process by helping coordinate activities between CHWs and supervisors [60–62], providing a lower-cost alternative to conventional face-to-face supervision [63], and making supervisory visits more personalized [50]. In Zambia, CHWs used simple-feature cell phones to submit weekly reports, order drugs and supplies, and send referral notices to health centres. Supervisors used cell phones to track CHW data, provide feedback to CHWs on referred patient outcomes, and receive monthly reminders to schedule supervision sessions with CHWs. A study found that these phone-based tools were both feasible and viable for the provision of real-time community-level information. However, additional support was needed to help CHWs overcome hardware and software challenges introduced by the new technology [60].

In the same region of Zambia several years later, a trial compared health centre catchment areas where CHWs and their health facility supervisors implemented mHealth-enhanced iCCM supervision and supply chain management against catchment areas implementing iCCM as per current Zambian guidelines. CHWs receiving the intervention used mobile phones for reporting and to requisition commodities. Supervisors received reports electronically, received monthly automated supervision reminders, and organized medical supplies for CHWs after receiving requisitions. The mHealth tools helped supervisors and CHWs manage supply chain issues. However, there was no impact on coverage of monthly supervision visits and no assessment of supervision visit quality [61]. The incremental cost of using the mHealth intervention per child contact for all iCCM conditions was US$ 11.35, and the largest share of costs (36%) was associated with supervision [62].

mHealth tools have also been used to change the format of supervision visits. A randomized control trial in Pakistan used technology to facilitate the training and supervision of CHWs, called lady health workers (LHWs), in the psychosocial management of perinatal depression. The study compared the competency of LHWs who received technology-based training and supervision from a distance versus those who received training and supervision in a conventional face-to-face format [63]. LHWs in both arms demonstrated competency improvements over time as a result of monthly supportive supervision, although the technology-assisted, distance-based approach was found to be 30% less expensive than the traditional face-to-face approach; outcomes of intervention delivery in the target population were not examined [63]. In Mali, dedicated CHW supervisors used a digital CHW performance dashboard to provide personalized feedback to CHWs during monthly supervisory visits; use of the dashboard significantly increased the mean number of home visits by almost 40 visits per month (*P* = 0.031) [50].

Our exploratory research did not include any formal evaluations of mHealth supervisory interventions in large-scale programmes; however, descriptive case studies from Nigeria, Rwanda, and Thailand suggest that mHealth tools are being employed to facilitate supervision in large-scale programmes. In Nigeria, some programmes have begun using short message service (SMS) to send informative messages to the mobile phones of VVHWs for the purpose of monitoring, mentoring, and improving performance [32]. In Rwanda, CHWs are equipped with a mobile phone and use the national RapidSMS programme to collect and submit data, thereby streamlining supervisors’ involvement in this task [34]. Lastly, in Thailand, where village health volunteers receive no formal supervision, a web-based information platform and mobile Android application has been introduced [38]. Unfortunately, we were unable to find rigorous evaluations of these mHealth interventions related to supervision in large-scale CHW programmes.

### Challenges of CHW supervision

Particularly for programmes operating at large scale, CHW supervision is especially challenging to implement effectively. Fifteen out of 29 of the large-scale programme case studies included in this exploratory research suffered from “weak supervision” [12]. Supervision was the fourth most commonly cited challenge among all the challenges mentioned in the case studies, behind lack of supplies, financing, and compensation [12].

Common supervision-related challenges identified in our exploratory research included lack of funds to pay for travel expenses and lack of transport for supervisory field visits, lack of clarity on supervisors’ roles, lack of appropriate tools and support to conduct supervision, lack of prioritization of supervision, supervisors’ poor understanding of the CHW’s role and context, frequent turnover of trained CHW supervisors, shortage of supplies with which to restock CHWs, and gender issues [12, 46, 49, 64, 65].

**Table:** 

Key message box 4	
The largest current single collection of case studies on large-scale CHW programmes demonstrates that 15 out of 29 of the programmes evaluated had “weak supervision” characterized primarily by lack of support (e.g., funds, tools, training, role clarity) for supervisors themselves	

These challenges point to the interdependencies between CHW supervision and other CHW programme elements such as community engagement, compensation, roles and responsibilities, necessities and supplies, financing, knowledge and skills training, motivation, and work environment (including integration with the national health system) [11, 46]. Efforts to enhance supervision without addressing other programme elements can fall short. For example, in a randomized control trial in Pakistan, supervisors of LHWs comprising the intervention arm received four days of supplemental training to improve their clinical and supervisory skills, including giving feedback to LHWs; the comparison group received standardized supervisory practices which included regular refresher training and a phone allowance for communicating with LHWs. Although the intervention improved supervisory performance, measured as provision of feedback to the LHW and setting clear expectations, the provision of commodities, a common supervisory responsibility in CHW programmes [10], was not addressed. Consequently, LHWs lacked commodities with which to provide care, resulting in only 18% of intervention arm caregivers reporting that LHWs were capable of providing diarrhoea and pneumonia care [66].

Supervision is a social concept that involves people and relationships. Consequently, it can be adversely impacted by strained relationships between CHWs and other actors who play a role in supervision, such as facility staff, community members, or peers [44, 67]. This is made more complex by the fact that CHWs often report to multiple supervisors working in different programmes [46]. Similarly, CHW supervisors must navigate multiple reporting lines, often at different levels of the health system that do not necessarily communicate with each other, and with other individuals who may not share the same understanding of CHWs’ purpose and role; these reporting lines impact factors related to interpersonal trust, which in turn can impact CHW performance [49].

Finally, although the costs of supervision vary significantly based on the programme and context, supervision is generally viewed as expensive [68]. In at least one study this was due to high supervisory personnel costs [69]. Certain approaches, specifically dedicated supervision, may carry additional personnel costs that make them even more challenging to sustain over time, as evidenced in the South African example in which the provincial government failed to renew the contracts of supervisors who were dedicated OTLs [49]. More studies are needed to measure the cost-effectiveness of different approaches**.**

## Discussion

Given the exploratory nature of this research, it would be inappropriate and infeasible to make universal recommendations about the best supervision approaches for CHW programmes. We found that the types of supervision approaches employed vary significantly by country and programme, a finding that is consistent with previous reviews of CHW supervision [11]. The variety of approaches makes it challenging to compare outcomes across programmes and to draw universal conclusions about what supervision approaches work best. Although CHW supervisors are expected to play a wide variety of roles, ranging from clinical oversight to administrative support to coaching and mentorship, none of the studies we reviewed explicitly examined supervisors’ ability to perform all of these roles, and what support would be needed to do so successfully. Another gap in the literature is the role of supervisors in hiring and firing CHWs, complicated by the fact that many CHWs currently operate as volunteers; this merits further research. Locally tailored solutions that leverage community structures and build on previous efforts to strengthen and reform large-scale CHW programmes are needed.

Our findings reveal that it is common for multiple supervision approaches to be employed in combination. Supervision is much more complex than a “dyadic relationship between the supervisor and supervisee”, with a number of different people carrying out a supervisory role [44]**.** Innovative combinations of CHW supervision approaches, implemented alongside other health systems support such as training and community engagement, are especially promising and merit further research.

**Table:** 

Key message box 5	
Innovative combinations of CHW supervision approaches, implemented alongside other health systems support such as training and community engagement, are especially promising and merit further research	

There are improvements in supervision in newly established programmes that are learning from the inadequacies of supervision in many older programmes. In large-scale programmes that have historically relied primarily on facility-based or district-level staff to supervise CHW cadres and volunteers, this approach is being supplemented with alternative approaches that rely on or leverage community committees, community members, and current or former peers [12, 70]. In addition, new tools have been developed to assess the supervision experience from the perspective of CHWs themselves, providing much-needed insight from the intended beneficiaries of supervision approaches [71]. While it is too soon to draw conclusions on the use of mHealth to enhance supervision, mHealth appears to improve communication between CHWs and supervisors which may improve, for instance, supervisors’ ability to ensure CHWs receive supplies ordered [61]. Additionally, mHealth interventions have been shown in at least one study to be as effective as traditional face-to-face methods but significantly more affordable [63].

A key challenge we identified in our results is the general lack of support for CHW supervisors, whether in the form of training, job aids, well-defined job descriptions, supervision of supervisors, funds, or access to transport, among others [49]. Cascade interventions that provide training and supervision for CHWs as well as management support for supervisors themselves show promise for addressing this challenge. These “cascade” interventions define who supervises the supervisors, their roles, and what tools are needed for this supervision to be effective. They also reinforce linkages between supervisors and the broader health system, enabling escalation and resolution of problems identified at the community level [57]. Such integration of CHW supervision with the broader health system can also help address contextual health system factors (e.g., incentives, availability of equipment and supplies, community engagement) upon which CHW supervision depends in order to positively impact CHW motivation and performance. Providing adequate management support for supervisors requires sustained funding founded on an a priori assumption that CHWs—just like other cadres of health professionals—deserve high-quality supervision that is well integrated at all levels of the health system.

This exploratory research did not include an assessment of the quality of included studies. We acknowledge that this limits readers’ ability to comment on the reliability of data and this review's conclusions. Future research that seeks to answer the question of what CHW supervision approaches work best and in what context should include a thorough analysis of the quality of included studies in order to inform future recommendations and guidelines. Another limitation of this study is its overwhelming reliance on case studies not subject to the academic peer review process. However, all of these case studies were authored by individuals with first-hand familiarity with the programmes or were drawn from key informants with such experience. As there is little systematized information about national CHW programmes, these case studies therefore provide critical data on CHW programme supervision otherwise missing from the readily accessible evidence base [12].

## Conclusion

Given this unique moment in which CHWs are at a critical threshold as the world moves towards UHC, and recognizing that UHC cannot be achieved without CHWs, CHW programmes now have the opportunity and the necessity for instituting stronger supervision as one of the critical steps in moving to a higher level of performance. Assessing quality and effectiveness of CHW supervision is key. In the absence of a single “best approach”, and in light of the limited evidence around optimizing CHW supervision, an emerging recommendation is that countries and programmes should prioritize homegrown evolution over time to suit the local context. In some cases, this may involve scaling up novel approaches that have proven effective at small scale or testing approaches that have worked in other countries. Increased, dedicated funding from national governments with support from the international community will be required to try new approaches, scale up existing approaches deemed promising, and evaluate the quality and effectiveness of such approaches alongside other health systems improvement initiatives.

Better monitoring systems and additional research is critical to improving supervision over time and identifying what works well in a particular context. Stronger evidence on how to make supervision more effective can help mobilize and direct new funding for CHW programmes and for supervision specifically, as well as make better use of current investments by optimizing supervision in existing CHW programmes. While the jury may still be out when it comes to specific approaches for CHW supervision, there is little doubt about the need to support both CHWs and their supervisors to do their roles to the best of their abilities. The still as yet unrealized full potential of CHWs to contribute to “Health for All”, as envisioned at Alma-Ata in 1978 [72], as well as to contribute to the Sustainable Development Goals [73], should inspire all stakeholders to continue the persistent pursuit of quality-improving practices, including supervision of CHWs, that are essential to help CHWs achieve their potential.

## Data Availability

Not applicable.

## Abbreviations

APE: *Agente polivalente elementar*
CHW: Community health worker
CHSS: Community health service supervisor
iCCM: Integrated community case management
LHW: Lady health worker
LMICs: Low- and middle-income countries
NGO: Nongovernmental organization
OTL: Outreach team leader
PHC: Primary healthcare
SMS: Short message service
UHC: Universal health coverage
WBPHCOT: Ward-based primary healthcare outreach team
